# Development of a Near Infrared Multi-Wavelength, Multi-Channel, Time-Resolved Spectrometer for Measuring Brain Tissue Haemodynamics and Metabolism

**DOI:** 10.1007/978-1-4939-0620-8_24

**Published:** 2014-03-22

**Authors:** Luke Dunne, Jem Hebden, Ilias Tachtsidis

**Affiliations:** Department of Medical Physics & Bioengineering, University College London, Malet Place Eng. Building, Gower Street, London, WC1E 6BT UK

**Keywords:** NIRS, TRS, Cytochrome-c-oxidase, Supercontinuum laser, Haemoglobin

## Abstract

We present a novel time domain functional near infrared spectroscopy system using a supercontinuum laser allowing us to measure the coefficient of absorption and scattering of up to 16 multiplexed wavelengths in the near infrared region. This is a four detector system that generates up to 3 mW of light for each wavelength with a narrow 2–3 nm FWHM bandwidth between 650 and 890 nm; each measurement of 16 wavelengths per channel can be performed up to a rate of 1 Hz. We can therefore quantify absolute haemoglobin changes in tissue and are currently investigating which and how many wavelengths are needed to resolve additional chromophores in tissue, such as water and the oxidation state of cytochrome-c-oxidase.

## Introduction

Near infrared spectroscopy (NIRS) is commonly used for non-invasive measurements of the concentration changes of oxyhaemoglobin (HbO_2_) and deoxyhaemoglobin (HHb) in tissue. Typically, continuous wave (CW) systems are used where a reflected/transmitted change in light attenuation through tissue is measured. If the scattering of light in the tissue is assumed constant and the differential path length factor estimated, the modified Beer–Lambert law can be used to calculate changes in chromophore concentrations [1]. CW systems have the benefit of requiring relatively simple and inexpensive components, and can be made into easy to use compact devices.

Time-resolved spectroscopy operates by pulsing short picosecond pulses of light into the tissue through optical fibres. Fast single photon detectors and highly accurate timing electronics are then used to measure the time-of-flight (TOF) of each photon escaping the tissue surface. By repeating this TOF measurement many times a histogram called a temporal point spread function (TPSF) can be generated. We can obtain much more detailed information about the tissue from the TPSF than is possible using a CW technique, including mean path length and the absolute absorption and scattering coefficients [2].

Advances in technology have reduced the cost and size of the timing electronics needed for TOF measurements, making the technique reasonably accessible. Time-resolved systems are therefore becoming increasingly popular for tissue diagnostics.

In addition to haemoglobin, cytochrome-c-oxidase (CCO) the terminal electron accepter of the respiratory chain is a strong absorber of near infrared light [3]. The absorption spectrum of CCO depends on whether the enzyme is in its oxidised or reduced state; NIRS utilises this to measure the changes in its oxidation state (oxCCO). Although there is a clear optical signature in the difference between the reduced and oxidised forms of CCO, the measurement of oxCCO is considerably more difficult than haemoglobin as the concentration in tissue is of an order of magnitude less [4]. Therefore, in order to decouple the haemoglobin and oxCCO changes accurately it is necessary to enhance the spectroscopic resolution of the NIRS system and measure independently absorption and scattering in many wavelengths [5]. CW broadband [6] and recently hybrid broadband and frequency domain systems have been used to measure oxCCO [7]. Zhu and colleagues using computational techniques and data from a CW broadband system during severe hypoxic-ischaemia in piglets have found that not only is the number of wavelengths important but there is significant improvement in the estimation of chromophores if specific combinations of wavelengths are used [8].

In order to address these issues we have designed and built a near infrared time domain multiwavelength spectrometer using a supercontinuum laser source. This enables us to measure the coefficient of absorption (μ_a_) and the reduced coefficient of scattering (μ_s_′) for 16 wavelengths between 650 and 890 nm. Here we describe the hardware of the system, discuss the theory of operation and present some preliminary results from the use of the system to monitor haemodynamic changes in the muscle during an arm cuff occlusion experiment.

## Instrumentation and Methods

**Fig. 24.1:**
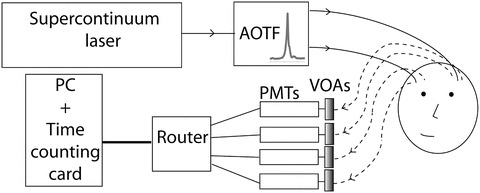
Schematic of time resolved multiwavelength near infrared spectrometer. The light is tuned using acousto-optic tunable filters (AOTF) and detected with four photomultiplier tubes (PMTs) each protected by variable optical attenuators (VOAs)

A custom designed supercontinuum laser (SC-480-6, Fianium, UK) with a repetition rate of 60 MHz producing white light over a range of 400–2,100 nm is used. The light is passed via optical fibre into a dual acoustic optic tunable filter (AOTF) system. As seen in Fig. 24.1, the light is collimated in free space and split by a polarising beam splitter. These beams are then passed through two AOTFs mounted at right angles to each other. As the devices only filter light in one plane this is the most efficient way of maximising the output power. The filters consist of piezo-electric transducers bonded to a birefringent quartz crystal that create a standing wave at a driven frequency. This modulates the refractive index of the crystal creating a phase grating, splitting the light from the laser into its different diffraction orders. A desired frequency can thus be directed and focused into an optical fibre. Custom AOTFs are used in the system to give narrow band filtered light of 2–3 nm FWHM in the region of 600–1,100 nm, these can provide an output power of 3 mW per wavelength.

**Fig. 24.2:**
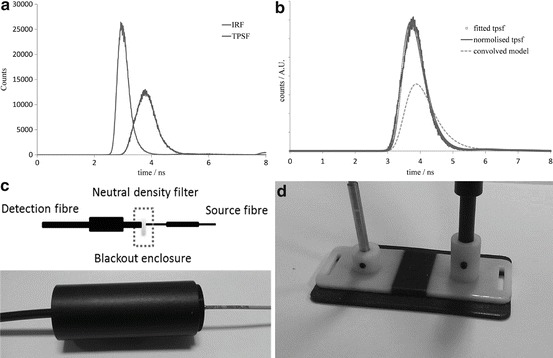
(**a**) Raw TPSF (scaled) and IRF, (**b**) theoretical model convolved with the IRF, the real TPSF and fitted curve using the lsqcurvefit function returning μ_a_ and μ_s_′ as 0.0224 and 1.0655 mm^−1^. (**c**) Method of measuring IRF. (**d**) 3D printed optode holder

For spectroscopy of tissue, each wavelength is multiplexed so that the TPSFs are averaged over the total desired measurement duration. The AOTF fast switches between 16 wavelengths at 160 Hz allowing time domain measurements with any combination of wavelengths between 650 and 890 nm. Two source fibres are used simultaneously when the detectors are placed on either hemisphere of the adult head. The light is passed into a 70 μm high NA single fibre which is attached to the patient via a custom designed 3D printed optode holder (Fig. 24.2d).

The light is collected by four glass fibre bundles (Loptek) with a diameter of 3 mm and is passed through custom made variable optical attenuators (VOAs) with a range of 0–3.7 OD to four Hamamatsu H7442-50P photomultiplier tube (PMT) modules. As the PMTs have a high gain the VOAs protect against over exposure during the experiment increasing the dynamic range. The signal from the PMTs is passed through a four way router (HRT-41) and the arrival time of each photon is measured with a Becker and Hickl SPC-130-EM time correlated single photon counting card.

The TSPF obtained in time resolved measurements contains information not only from the tissue but also the instrument itself. Therefore, a correction has to be made before the true optical properties of the tissue can be obtained. An instrument response function (IRF, Fig. 24.2a) is recorded before each measurement using neutral density filters (Fig. 24.2c) in order to characterise the factors which contribute towards the broadening of the IRF (laser pulse, optical fibres, photon detectors, and timing electronics).

In order to quantify the optical properties of the tissue, the solution to the diffusion equation for a semi-infinite homogenous medium was convolved with the IRF (Fig. 24.2b) [9]. The convolved model is fitted to the measured TPSF using a non-linear curve fitting function and the absorption coefficient, μ_a_ and reduced scattering coefficient, μ_s_′ obtained. The Beer–Lambert law was then used to calculate chromophore concentrations [10]. To test the hardware and theory of operation we performed an arterial cuff-occlusion on the upper arm in one volunteer to induce flow and oxygenation changes in the forearm flexor muscles. The probe was placed on the forearm and measurements were done in reflection mode with source and detector fibres 3 cm apart. After 100 s of baseline measurements we inflated the cuff at 200 mmHg for 300 s, following cuff deflation we continue monitoring the muscle recovery for 5 min.

Data were collected every second for eight common wavelengths used in near infrared spectroscopy [690 750 761 790 801 834 850 870]. The average count rate over the experiment was kept at over 10^6^/s to provide a good enough SNR for each wavelength. The diffusion equation model was then fitted to each TPSF to resolve absorption and scattering.

## Results

**Fig. 24.3:**
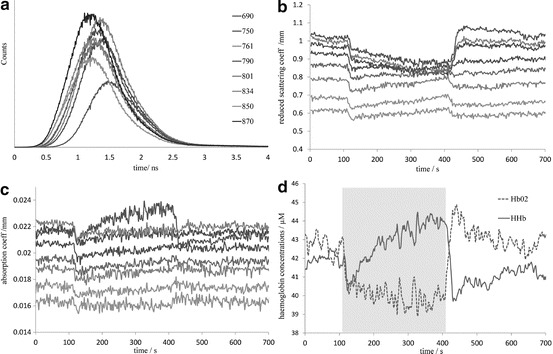
(**a**) Example TPSFs for single measurement. (**b**) Reduced coefficient of scattering for eight wavelengths over course of cuff occlusion. (**c**) Coefficient of absorption for eight wavelengths over course of cuff occlusion. (**d**) Concentration changes for HbO_2_ and HHb, cuff inflation time of 4 s

Time series data for the changes in scattering and absorption for all eight wavelengths are shown in Fig. 24.3b, c, respectively. The standard deviation of the scattering and absorption during baseline was 0.0002 mm^−1^. During the occlusion there were large changes in the absorption in some wavelengths, in particular there was a significant rise in absorption of 690 nm (sensitive to HHb) and significant decreases in 870 nm (sensitive to HbO_2_). The scattering also demonstrated some hetereogeneous large changes and we are currently investigating whether these might be due to crosstalk or other factors. Finally, the absorption data were fitted for HbO_2_ and HHb, the baseline total haemoglobin was 84.8 ± 0.3 μM l^−1^ and the absolute tissue saturation was 51.8 ± 0.5 %, comparable to previous studies in muscle [11]. Figure 24.3d shows the absolute concentration of oxyhaemoglobin and deoxyhaemoglobin during the study.

## Conclusion

We have developed a four-channel NIR time-resolved spectrometer using a supercontinuum laser source and tunable narrow band filter system capable of measuring the TPSFs of 16 wavelengths between 650 and 890 nm every second in order to quantify the scattering and absorption independently for tissue. This offers us the ability to extract changes in haemoglobin and other chromophores in tissue such as CCO. We have presented preliminary results of the operation of the system for one channel and eight wavelengths during an arm cuff occlusion test. We are currently using the system to investigate wavelength selection optimisation for resolving HbO_2_, HHb and oxCCO and will be carrying out a series of functional activation studies.
